# Genetic rules for the dermatoglyphics of human fingertips and their role in spouse selection: a preliminary study

**DOI:** 10.1186/s40064-016-3072-x

**Published:** 2016-08-22

**Authors:** Xiao Yang, Jin Xiaojun, Zhou Yixuan, Liu Hui

**Affiliations:** College of Medical Laboratory, Dalian Medical University, Dalian, 116044 China

**Keywords:** Dermatoglyphics, Fingerprints, Whorls, Married couple, Children, Genes

## Abstract

**Objective:**

We assessed the genetic rules for the dermatoglyphics of human fingertips. We also evaluated the correlation between spouse selection with the number of whorls on fingertips.

**Methods:**

Data were collected for the number of whorls from 118 families (couples and their children). Distribution of whorls was analyzed further to investigate the relationship between heredity and spouse selection.

**Results:**

Through multiple regression analysis, we found that the number of whorls on fingertips was affected considerably by genetic factors. In a married couple with a moderate number of whorls, the probability of their children having a high number of whorls was 26.5 %, and the probability of their children having a low number of whorls was 23.5. These values were close to the theoretical value (25 %). A significant correlation between whorl count between spouses was observed.

**Conclusion:**

These data suggest that whorls are inherited from a single gene or a group of closely linked genes. Our findings provide an initial insight on the potential contribution of biologic characteristics on spouse selection.

## Background

A “fingerprint” is an impression left by the friction ridges of a human finger. “Dermatoglyphs” are skin patterns (in particular patterns of the specialized skin of the inferior surfaces of the hands and feet). “Dermatoglyphics” is the scientific study of fingerprints (Morgan 1979).

A fingertip dermatoglyph is formed in the embryonic phase, and is a unique characteristic in humans and non-human primates (Gutiérrez et al. 2007; Wijerathne et al. 2013). There are three basic fingerprint patterns: “loop”, “whorl” and “arch”, which constitute 60–65, 30–35 and 5 % of all fingerprints, respectively (Morgan 1979; Wijerathne et al. 2013). Various fingerprints tend to have a regular shape, such as round (whorl). However, the factors affecting the development of fingerprints towards a regular shape (e.g., size, thickness of subcutaneous fat, degree of stoutness, growth rate of bone) eventually result in the diversity of fingerprints. In general, it is thought that the dermatoglyphic pattern of a human fingertip (whorl, loop or arch) is controlled mainly by genetic factors (Solhi et al. 2010; Cheng et al. 2009; Dipierri et al. 2014), and that its diversification also reflects the genetic diversity of individuals (Madan et al. 2011).

Although dermatoglyphic pattern is a complex pleiotropic phenotype, to which a hitherto unknown number of genes contribute by interacting with each other and the environment, we still consider that there is one set of closely linked genes that are of primary importance in determining the dermatoglyphic traits. Our research team have hypothesized that all types of fingerprints may be derived from changes in the regular round fingerprint. According to this hypothesis, in the present study, we undertook genetic analyses of regular whorl-shaped fingerprints and revealed the related genetic rules.

Several reports on the genetic diversity of individuals with respect to fingerprints have focused on the association between fingerprints and various diseases (Sontakke et al. 2013; de Bruin et al. 2012; Bukelo et al. 2011). Unexpectedly, we also found that the number of whorl-shaped fingerprints of a husband was correlated to that of his wife. This observation suggested that the number of whorl-shaped fingerprints of a person will influence his/her selection of a spouse. This is the first time such a phenomenon has been reported.

Spouse selection was determined by examining social factors (Samani 2007; Samani and Ryan 2008; Basavarajappa et al. 1988; Manfredini et al. 2010) and biologic factors (Manfredini et al. 2010; Silventoinen et al. 2003; Knuiman et al. 2005). The latter included apparent and non-apparent features: body height; appearance and genetic characteristics. However, there was limited information available to distinguish the non-apparent factors when evaluating spouse selection. Therefore, the mode of inheritance of whorl-shaped fingerprints and the relationship between the number of whorl-shaped fingerprints and spouse selection requires further attention.

## Methods

### Ethical approval of the study protocol

The Institutional Ethics Committee of Dalian Medical University approved the study and waived the need for written informed consent from the participants due to the observational nature of the study.

### Subjects and collection of fingerprints

Chinese university students (age 18–22 years) and their biologic parents were enrolled as subjects. Each student and each pair of parents were treated as one family. The husband and wife were unrelated ethnic Han Chinese individuals from urban or rural random population in China. All subjects did not pay special attention to their fingerprints for any purpose.

Fingerprints were collected using an ink-imprinting method. If collected fingerprints were not clearly defined, the corresponding family was removed from the enrolment. A total of 118 families (46 males and 72 females) formed the study cohort.

### Definition and discrimination of a regular “whorl-shaped” fingerprint

If a round or oval fingerprint had a clear central point, dermatoglyphic ridge lines were clear and regular, and no irregular changes were present in a wide range (number of ridge lines >10), then such a fingerprint was determined to be “regular whorl-shaped”. A “double loop whorl” nor “multiple-loop whorl” was not determined to be “regular whorl-shaped”. Our assumption was that various types of fingerprints are derived from changes in a regular whorl-shaped pattern. Hence, fingerprints were divided into two categories: regular whorl-shaped pattern and “other”.

Personnel assessing fingerprint samples were fully trained and familiar with the criteria that we used. Each fingerprint sample was assessed by three persons independently. The result was documented if at least two of the assessors came to the same conclusion.

### Statistical analyses

The sum of whorls on the ten fingers for each person was calculated. The effect of the number of whorls of the parents on that of their child was analyzed using multiple regression analyses. If the multiple regression equation was significant, the indicator was considered to be influenced by genetic factors, the degree of which was measured using *R*^2^.

To observe the symmetry between whorls on a left finger and that on the corresponding right finger, subjects with total of two or four whorls were selected for the test. This strategy was used to ensure that the distribution of whorls on ten fingers had sufficient degrees of freedom, and that symmetric distribution of whorls between a left finger and the corresponding right finger was observed. In theory, the sum of symmetric finger pairs should be half the total number of whorls. The degree of similarity between the theoretical value and the observed value was reviewed using a binomial test.

**Fig. 1:**
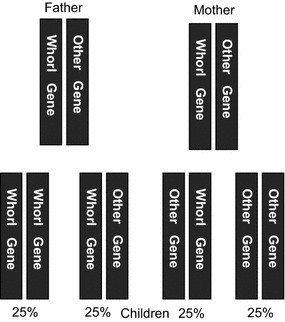
Theory of the rule of inheritance of whorls. Subjects with a moderate number of whorls had one “whorl gene” and one “other gene”. Thus, for a married couple with a moderate number of whorls, the probability of their children having a high number of whorls (8–10) should be 25 % (whorl gene and whorl gene); the probability of their children having a low number of whorls (0–2) should also be 25 % (other gene and other gene)

To assess the rule of inheritance for whorls, a husband and his wife, each with a moderate number of whorls (3–7), were selected. If 1/4 of their children had a high number of whorls (8–10) and 1/4 of them had a low number of whorls (0–2), this finding suggested that whorls were inherited from a single gene or a group of closely linked genes (Fig. 1). The degree of similarity between the theoretical value and observed value was reviewed using the Chi squared test.

Correlation between the number of whorls of a father and that of the mother (a husband and his wife) was also analyzed.

Statistical analyses were performed with the SPSS statistical analysis software (SPSS, Chicago, IL, USA). A difference was considered to be statistically significant when the *P* value was <0.05 (using a two-tailed test).

## Results

For assessment of the correlation between the number of whorls of children and those of their parents, the following multiple regression equation was obtained:

$$ {\text{C }} = \, 0.249{\text{F }} + \, 0.33{\text{M }} + \, 1.914 \, \left( {{\text{R}}^{2} = \, 0.259,{\text{ P}} < 0.001} \right) $$

where C, F and M represent number of whorls in the child, father and mother, respectively.

A total of 33, 28 and 30 subjects in the groups of children, fathers and mothers had two or four whorls, respectively. Paired theoretical and observed values are shown in Table 1. Theoretical values were significantly different from observed values, suggesting that whorls were distributed randomly on ten fingers rather than distributed in a symmetric pattern.

**Table 1:** Observation of symmetry between whorls on a left finger and that on the corresponding right finger

Group	Total fingers	Paired fingers		*P*
		Expected	Observed	
Child	88	44	22	<0.001
Father	84	42	23	<0.001
Mother	88	44	23	<0.001

Thirty-four husband-and-wife pairs each with a similar moderate number of whorls were selected. A high number and low number of whorls in their children were consistent with theoretical values (Table 2), suggesting that the mode of inheritance of whorls was based on a single gene or a group of closely linked genes.

**Table 2:** Observed and expected percentages of a high, middle and low number of whorls in children from 34 parents with a moderate number of whorls (3–7 whorls)

Groups of children with number of whorls	Number	Observed (%)	Expected (%)	*P*
High (8–10)	9	26.47	25.00	
Medium (3–7)	17	50.00	50.00	0.971
Low (0–2)	8	23.53	25.00	
Total	34	100.00	100.00	

Initial data for a husband-and-wife pair with respect to the number of whorls is given in Table 3. Correlation analyses suggested that the number of whorls on ten fingers of a husband was significantly correlated to that of his wife.

**Table 3:** Correlation between the number of whorls between a husband and his wife (n = 118)

Number of whorls	
Husband	Wife (mean ± SD)
0	0.60 ± 1.27
1	3.25 ± 2.99
2	2.93 ± 1.82
3	3.42 ± 2.78
4	4.21 ± 3.04
5	5.40 ± 2.35
6	4.82 ± 2.93
7	3.88 ± 2.42
8	6.80 ± 1.93
9	6.50 ± 4.73
10	6.25 ± 3.01
*r*	0.486
95 % confidence intervals	0.355–0.665
*P*	<0.001

## Discussion

The main problem in determination of the mode of fingerprint inheritance is the diversity of the phenotype: the fingerprint of each person is uniquely different from that of another person. Hence, classification of the phenotype of a fingerprint can be inaccurate or subjective. Our research work has led us to assume that fingerprints tend to have a regular round shape. Since the factors affecting development of fingerprints towards regular round also influence the forming process of regular round fingerprints, this eventually leads to diversity of fingerprints. We assessed the genetic rules governing fingerprint patterns to clarify this problem.

We found that the number of whorls of the parents had a significant impact on the number of whorls of the child. This finding suggested that the number of whorls on fingers was associated with inheritance, and that our classification method was applicable to the genetic research of fingerprints. Further study also indicated that whorls were distributed randomly on ten fingers instead of a symmetric distribution on a left finger and the corresponding right finger. Thus, we further assumed that fingerprints on ten fingers are controlled by the same gene locus that has two alleles: “whorl gene” (W) and “other gene” (O). Results showed that for a husband and his wife each with similar moderate number of whorls, 1/4 of their children had a high number of whorls and that 1/4 of their children had a low number of whorls. These results were consistent with theoretical values, suggesting that gene W and gene O are dominant genes.

Unexpectedly, we found that the number of whorls on a husband’s fingers was significantly correlated with that on his wife’s fingers; the racial influence could be limited because all of the subjects were unrelated ethnic Han Chinese individuals in our study; therefore, it is suggested that the number of whorls on fingers has an influence on the selection of a mate. External genetic factors (e.g., height) can affect the selection of a mate, which may be attributable mainly to the role of social factors. However, the effects of genetic factors on selection of a mate has not been reported. Effects of genetic factors on selection of mates are perhaps derived from biologic factors, so their biologic significance may be more important. The findings of the present study may provide a good basis for other, similar studies.
